# The Sunrise of Tertiary Lymphoid Structures in Cancer

**DOI:** 10.1111/imr.70046

**Published:** 2025-07-01

**Authors:** Juliette Rochefort, Gilles Marodon, Jean‐Luc Teillaud, Marie‐Caroline Dieu‐Nosjean

**Affiliations:** ^1^ Inserm U1135 Paris France; ^2^ Sorbonne University, UMRS1135 Paris France; ^3^ Laboratory “Immune Microenvironment and Immunotherapy”, Center of Immunology and Microbial Infections (Cimi), Faculty of Health Sorbonne University Paris France; ^4^ Health Faculty, UFR Odontologie University of Paris Paris France; ^5^ Department of Odontology Pitié Salpêtrière Hospital, Assistance Publique‐Hôpitaux de Paris (AP‐HP) Paris France; ^6^ Department of Odontology Paris Cité University Paris France

**Keywords:** biomarker, humanized mouse, microbiota, sensory nerve, sympathetic nerve, tertiary lymphoid structure

## Abstract

First considered as a negative epiphenomenon in autoimmune and inflammatory diseases, with possible deleterious consequences through the production of pathological autoantibodies and antiself T cells, tertiary lymphoid structures (TLS) have gained major scientific and clinical interest in cancer due to their association with better clinical outcomes and improved responses to immunotherapy. Studies have investigated the structure and plasticity of TLS in the context of tumors and the role of the TLS B‐cell compartment in contributing to the favorable clinical outcome of cancer patients. Identifying biomarkers that indicate the presence of TLS in tumors in a noninvasive manner could therefore represent a major advance in the diagnosis and treatment decision‐making for these patients. Also, the interplay between TLS, tumor cells, and microbiota opens new avenues for deciphering the role of microorganisms in cancer development. Their use as TLS inducers further underlines the need for continued research in this field. Moreover, emerging data have emphasized the critical role of sensory and sympathetic nerves in regulating TLS formation and function. Finally, humanized mice may serve as valuable tools for developing preclinical models to study the role of human TLS in cancer, a much‐needed goal. These different topics are discussed in the present review.

## 1 Introduction: The Overwhelming Rise of TLS in Cancer

Lymphoid aggregates resembling lymph nodes (LNs) were first noticed in inflamed rheumatoid synovial tissue [1], and a decade later in thyroid tissue of patients with Hashimoto's thyroiditis, a severe autoimmune disease [2]. Following similar observations in other inflammatory and autoimmune conditions, these lymphoid aggregates were termed “tertiary lymphoid organs” by Louis Picker and Eugene Butcher [3]. They were later also referred to as “ectopic lymphoid tissues” [4] or “tertiary lymphoid structures” when the presence of germinal centers (GCs) in these structures was formally demonstrated [5]. The process of their formation was termed “lymphoid neogenesis” [6].

**FIGURE 1:**
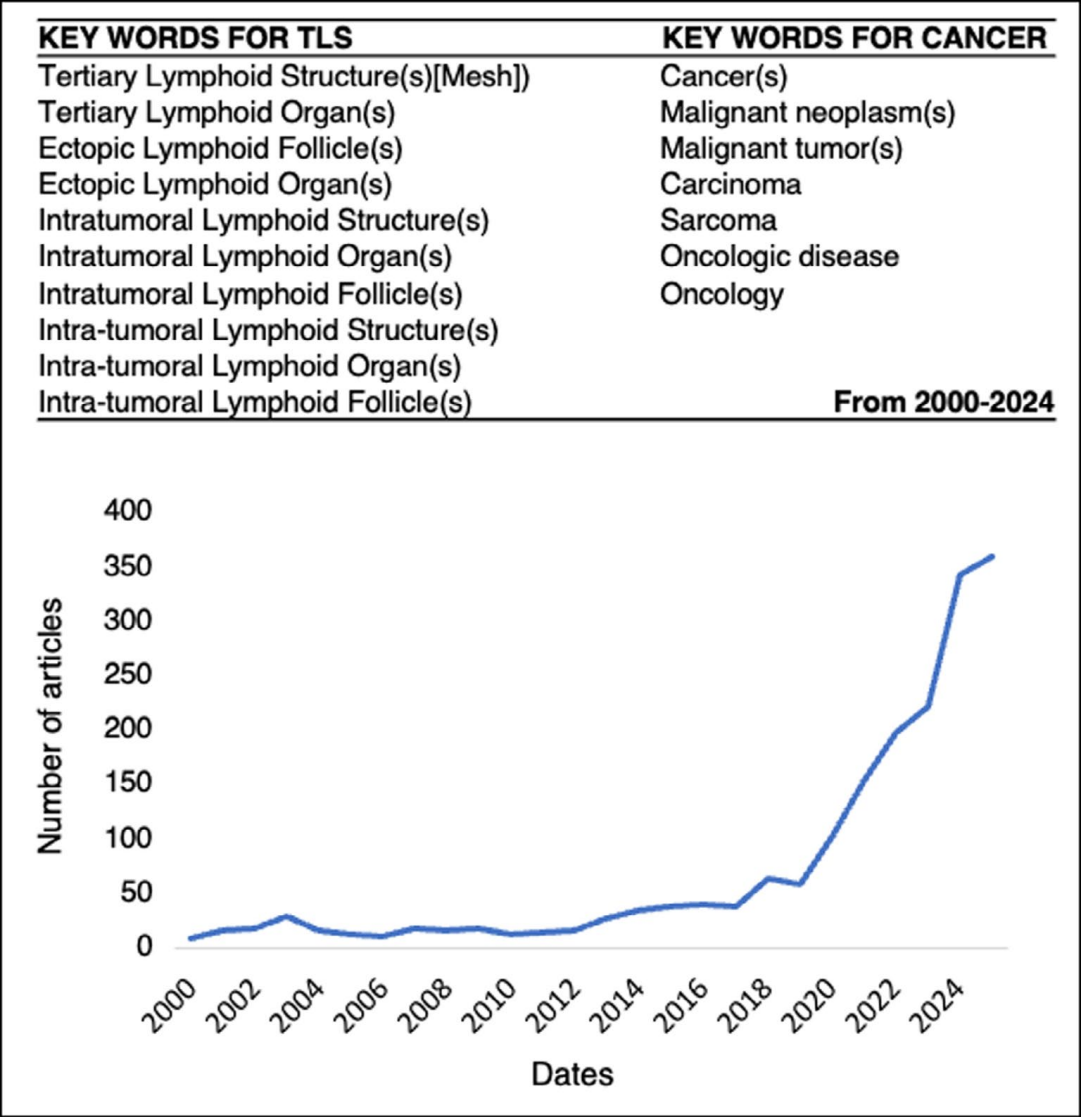
Trends in the number of publications on TLS in the context of cancer (2000‐2024). Annual number of scientific publications indexed in PubMed between 2000 and 2024, using the following search queries: (((Tertiary Lymphoid Structures[Mesh]) OR (Tertiary Lymphoid Structure) OR (Tertiary Lymphoid Structures) OR (Tertiary Lymphoid Organ) OR (Tertiary Lymphoid Organs) OR (Ectopic Lymphoid Follicle) OR (Ectopic Lymphoid Follicles) OR (Ectopic Lymphoid Organ) OR (Ectopic Lymphoid Organs) OR (Intratumoral Lymphoid Structure) OR (Intratumoral Lymphoid Structures) OR (Intratumoral Lymphoid Organ) OR (Intratumoral Lymphoid Organs) OR (Intratumoral Lymphoid Follicles) OR (Intratumoral Lymphoid Follicle) OR (Intratumoral Lymphoid Structure) OR (Intratumoral Lymphoid Structures) OR (Intratumoral Lymphoid Organ) OR (Intratumoral Lymphoid Organs) OR (Intratumoral Lymphoid Follicles) OR (Intratumoral Lymphoid Follicle))) AND (((cancer) OR (cancers) OR (malignant neoplasm) OR (malignant neoplasms) OR (Malignant tumor) OR (Malignancy tumor) OR (carcinoma) OR (sarcoma) OR (Oncologic disease) OR (Oncology))) Filters: From 2000 to 2024.

The first description of tertiary lymphoid structures (TLS) and their prognostic value in cancer was reported in 2008 in non‐small‐cell lung cancer (NSCLC) (termed in this study tumor‐induced bronchus‐associated lymphoid tissues (Ti‐BALTs)) [7]. Then, the number of articles and reviews devoted to these ectopic lymphoid structures in cancer has increased over the years until today (Figure 1). This trend has markedly intensified since 2020. Remarkably, this surge coincides with findings showing that TLS also serve as a predictive biomarker of patient response to targeted therapies.

Thus, to avoid duplicating the numerous reviews recently published on TLS in cancer, we will primarily present and discuss some emerging topics in this field. After summarizing the current debate on the nature of TLS and their role in cancer patients and cancer therapies, we will present some aspects of the current knowledge on their relationship with microbiota in cancer. We will also discuss whether TLS presence in solid tumors can be detected in a noninvasive manner through the analysis of body fluids. Then, their relationship with sensory and sympathetic neurons, in comparison to secondary lymphoid organs (SLOs) will be debated. Finally, we will also explore whether the use of humanized mice represents a promising approach to improve our knowledge on human TLS neogenesis and function in cancer.

## 2 Tertiary Lymphoid Structures in Cancer Patients

**FIGURE 2:**
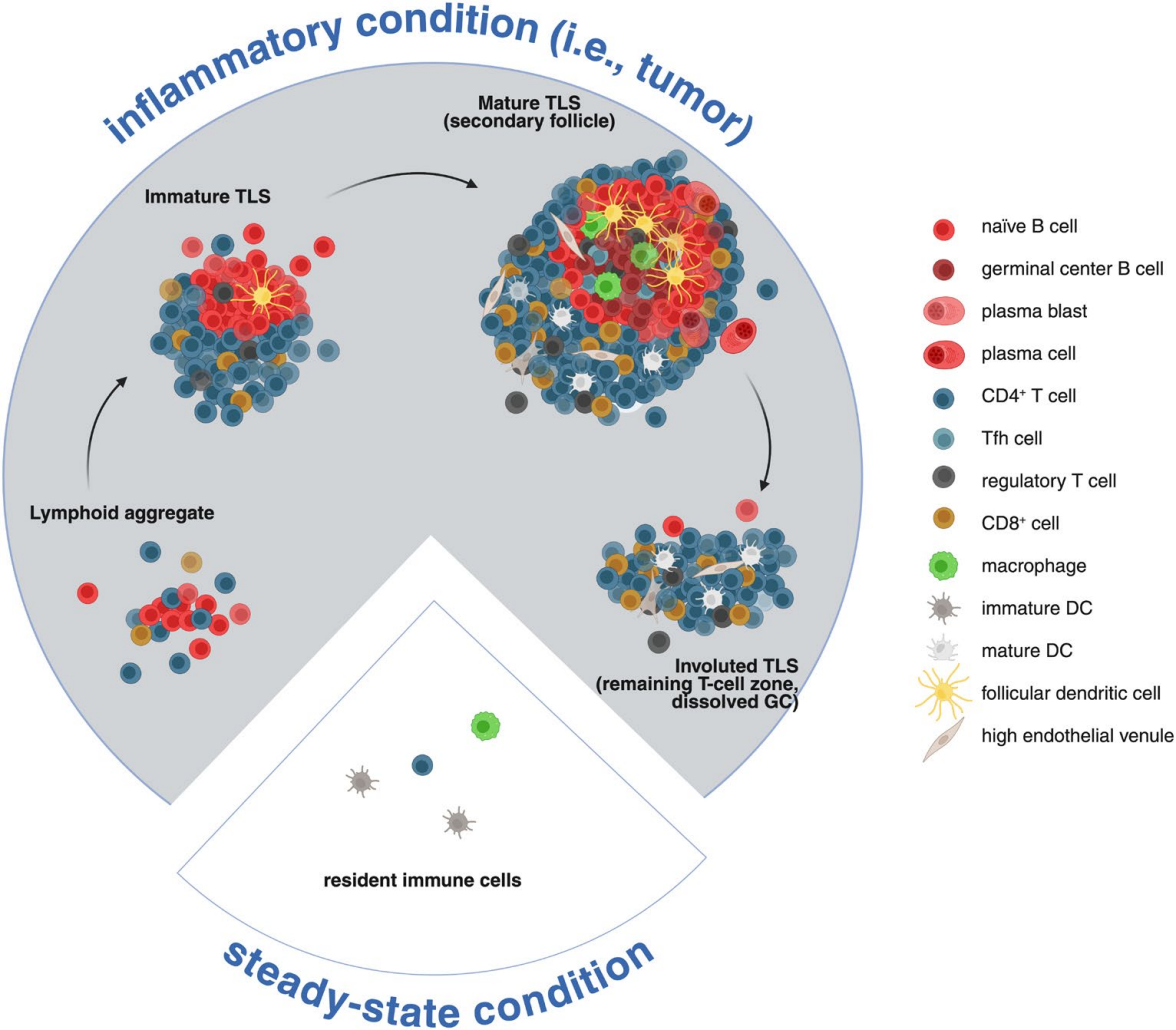
Evolution of the immune infiltration and organization in steady‐state versus inflammatory conditions, as illustrated in the tumor microenvironment. In a normal situation, some immune cell subsets reside in the tissue. Upon inflammation, an influx of immune cells arises in the tissue, such as through high endothelial venules (HEVs), and different levels of immune cell organization can be observed, from lymphoid aggregates, immature TLS to mature TLS, the most sophisticated state. Upon resolution of the inflammation, TLS involute with first, the regression of the B‐cell zone. Created in https://BioRender.com.

**FIGURE 3:**
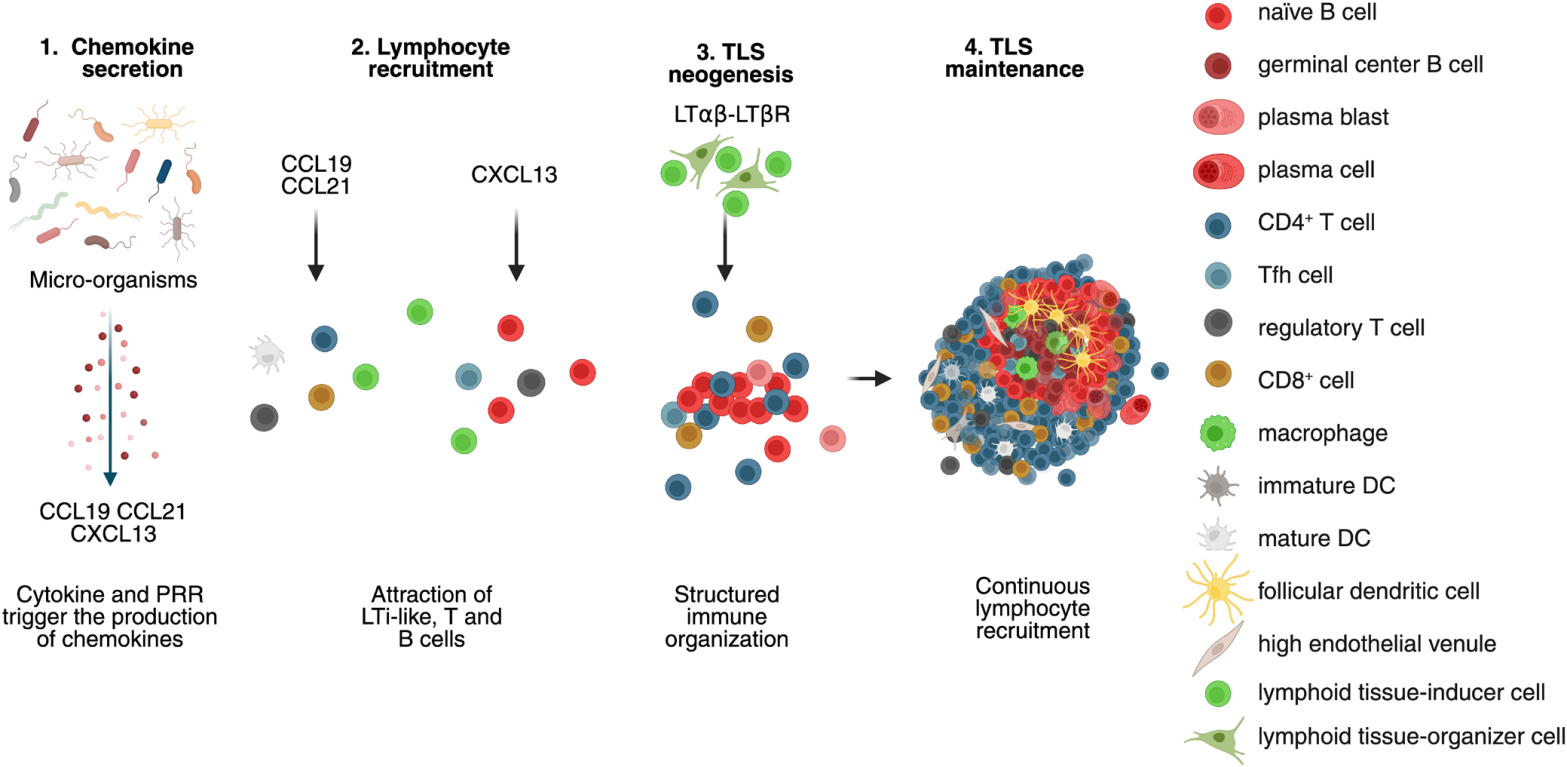
Bacteria release pathogen‐associated molecular patterns (PAMPs), which activate signaling pathways such as Mitogen‐activated protein kinases (MAPK), Janus kinase/signal transducers and activators of transcription (JAK‐STAT), and nuclear factor‐kappa B (NF‐кB), that stimulate the secretion of cytokines and chemokines conducive to TLS maturation. Created in https://BioRender.com.

Tertiary lymphoid structures are inducible ectopic lymphoid structures that develop and are maintained in response to chronic inflammation. Upon resolution of inflammation, TLS regress to return to a steady‐state condition. They exhibit the same cellular organization and composition as canonical LNs. They consist of a T‐cell rich area containing predominantly T cells and mature DCs, adjacent to a B‐cell rich area comprising mainly B cells, follicular dendritic cells (FDCs), follicular helper T cells (Tfh), and macrophages [8]. However, TLS lack NK cells in contrast to SLOs [9]. Several stages of TLS development have been reported in human tumors. At least four distinct stages can be defined based on their cellular density and composition, each likely corresponding to a specific stage of lymphoid neogenesis (Figure 2). The earliest stage, characterized by minimal organization, consists of loose lymphoid aggregates containing few DCs but lacking FDCs. The intermediate stage, referred to as immature or primary follicle‐like TLS, exhibits higher densities of T and B cells segregated in distinct zones, along with the presence of a FDC network; however, GCs are still absent. Fully mature TLS, also known as secondary follicle‐like TLS, display active GCs and high endothelial venules (HEVs), and are functionally competent in promoting T and B cell activation. This T–B cell segregation is orchestrated by the localized expression of lymphoid chemokines: the couple CCL19‐CCL21/CCR7 guides T cells and mature DCs to the T‐cell zone, while CXCL13/CXCR5 directs B and Tfh cells to the B‐cell zone (Figure 3). Finally, the resolution of the inflammation is closely linked to the regression of TLS (Figure 2), as nicely shown in hepatocellular carcinoma (HCC) following neoadjuvant anti‐PD1 treatment [10], and in a murine model where a transient inflammation was elicited upon intranasal LPS instillation [11].

The cellular composition and organization of TLS make them a potent immunological hub for initiating and/or reactivating adaptive immune responses at the effector site, which can ultimately be relayed to SLOs. Indeed, in several cancer types, all stages of T and B cell differentiation in TLS have been reported, from naïve to effector‐memory cells (i.e., central‐memory and effector‐memory cells for the T‐cell compartment, and memory and antibody‐secreting plasma cells for the B‐cell compartment). The immune function of TLS has been formally demonstrated in mice devoid of any SLOs, as illustrated in a model of Influenza virus infection [12, 13]. In this setting, the authors observed TLS formation in inflamed lungs and detected robust T and B cell responses as well as primary and memory immunity against the Influenza virus. In cancer patients, high densities of TLS were associated with long‐term survival in most solid tumors, that is, lung, breast, colorectal, head and neck, pancreatic, stomach carcinomas, and melanoma, as reviewed elsewhere [8]. Interestingly, each area of TLS, the T‐cell and the B‐cell zones, correlated with a favorable clinical outcome, as exemplified in NSCLC [7, 14], indicating that the cellular and humoral immune responses contribute to prolonged survival. It is worth noting that when combined, the two areas of the TLS better predict the outcomes of the patients, suggesting a coordination of the immune responses taking place in TLS. One may speculate that T and B cells may recognize the same set of tumor‐associated antigens. However, some studies have associated TLS with a poor prognosis. In clear cell renal cell carcinoma (ccRCC), genomic analyses revealed that tumors‐positive TLS exhibited a significant higher frequency of alterations in the PI3K‐mTOR pathway compared to TLS‐negative tumors. Notably, TLS^+^ patients experienced worse clinical outcomes than those without TLS [15, 16].

Finally, several studies have reported that high densities of TLS (especially determined through B‐cell signature) predict response to immunotherapy, as shown in urothelial, melanoma, ccRCC, sarcoma, NSCLC [17, 18, 19, 20, 21]. A retrospective analysis of cancer patients treated with anti PD‐1/PD‐L1 therapies revealed that the predictive value of TLS is independent of CD8^+^ T cell infiltration [22]. In muscle‐invasive bladder cancer, elevated CXCL13 expression correlated with TLS presence and improved outcomes following anti‐PD‐L1 therapy, irrespective of CD8A expression level, but was associated with the B‐cell marker CD19 [23]. Also, a high density of TLS‐CD22^+^ ADAM28^+^ B cells predicts response to anti‐PD‐1 treatment in NSCLC patients [24]. Collectively, these results suggest that the capacity of TLS to predict immunotherapy response may be at least mediated by B‐cell immunity.

## 3 Tertiary Lymphoid Structures in Infection‐Associated Cancers

The importance and respective roles of B and T cell compartments in TLS function have been investigated in cohorts of patients with various types of cancers [14, 17, 18, 25, 26, 27, 28, 29, 30, 31, 32]. However, one has to remind that TLS were initially described and studied in inflamed tissues of patients with autoimmune diseases [33, 34, 35], chronic allograft rejection [36], or infections such as hepatitis C virus (HCV) [37], influenza virus [12, 13], *Helicobacter pylori* [38], *Borrelia burgdorferi* (Lyme disease) [39], *Staphylococcus aureus* and *Pseudomonas aeruginosa* [40], among others as reviewed elsewhere [41, 42]. Interestingly, multiple microbial agents have been implicated in tumor initiation or promotion, but the relationship between tumor development, microbial infections, and TLS neogenesis remains barely understood. Microorganisms exert multifactorial influences on tumorigenesis. Bacteria, parasites, viruses, and fungi are all active subjects of investigation for their potential roles in oncogenic processes [43].

Indeed, multiple microbial agents have been implicated in tumor initiation or promotion (Table 1). Some pathogens, including certain bacteria, parasites, and viruses, are now classified as established carcinogens [65]. Others, while not directly oncogenic, show significant associations with tumor progression. Conversely, some microorganisms exhibit protective effects by inhibiting tumor growth (Table 1). For instance, reuterin, an antimicrobial compound produced by *Lactobacillus reuteri* , inhibits colorectal cancer growth [58]; *Streptococcus thermophilus* has demonstrated immunomodulatory and antitumor properties [59]; and genera such as *Bacteroides* and *Parabacteroides* are associated with reduced melanoma growth in mice [59]. Moreover, microbial metabolites such as polyamines [58] and short‐chain fatty acids (SCFAs) [62, 63] can also modulate antitumor immune responses.

**TABLE 1:** Microorganisms and metabolites involved in cancer: Oncogenic, synergistic, or protective roles.

Microorganism or metabolite	Associated cancer	Microorganisms effect	References
**Oncogenic microorganisms**			
*Helicobacter pylori*	Gastric cancer MALT Lymphoma	NF‐kB/RASAL2/β*‐catenin*	[44, 45]
Schistosomiasis	Bladder cancer	Not indicated	[46]
*Clonorchis sinensis*	Cholangio‐carcinoma	Not indicated	[46]
Human papillomavirus (HPV)	Genital/cervical Tumor HNSCC	via E6 and E7, and also E2 and E5	[47, 48]
Epstein–Barr virus (EBV)	Nasopharynx carcinoma Gastric cancer Lymphoma	Not indicated	[49]
**Microorganisms contributing indirectly to cancer development**			
*Escherichia coli*	Nonspecific	Promote tumor progression by producing the toxic metabolite colitoxin	[50]
*Fusobacterium nucleatum*	Intestinal cancer	Activation of glycolysis and cell proliferation by upregulating specificity protein 1 (SPI) and selectively targeting specific enolasel‐intronic transcript 1	[51]
*Fusobacterium nucleatum*	Colorectal cancer	Induction of alpha‐kinase 1 (ALPKl) to stimulate the NF‐KB pathway, leading to the upregulation of intercellular adhesion molecule‐1 (ICAM‐1)	[52]
*Fusobacterium nucleatum*	Breast cancer	Induction of lymphocyte apoptosis through lectin Fap2, resulting in tumor growth	[53]
*Malassezia*	Pancreatic cancer	Accelerated tumor progression through the activation of mannose‐binding lectin (MBL); driving of IL‐33 secretion to recruit and activate Th2 and innate lymphoid cells 2 (ILC2); stimulation of IL‐13 secretion, and ultimately promotion of tumor growth	[54, 55]
*Blastomyces*	Lung cancer	Association with inflammation and metastasis	[56]
*Candida*	Gastrointestinal tumor	Association with inflammation and metastasis	[56]
*Phaeosphaeriaceae Phaeosphaeria*	Ovarian cancer	Related to shortened progression‐free survival	[57]
*Capnodiales Cladosporium*	Metastatic melanoma	Significant increase in patients who have no response after immunotherapy	[57]
**Microorganisms contributing to cancer prevention**			
Reuterin	Colorectal cancer	Protective effect and inhibition of tumor progression by altering redox balance and metabolite exchange	[58]
Bacteroides Parabacteroides	Melanoma in Rnf5^−/−^ mice	Inhibition of tumor growth modulation of DCs activation	[59]
*Streptococcus thermophilus*	Colorectal cancer	Promotion of apoptosis of tumor cells, thereby acting as a tumor suppressor	[60]
Polyamines	Liver cancer	Blockade of lymphocyte proliferation and stimulation of tumor‐associated proteases production	[61]
Lipoteichoic acid	Liver cancer	Stimulating the production of prostaglandin E2 (PGE2), leading to inhibition of antitumor immunity	[61]
SCFAs	Nonspecific	Enhance the antitumor activation of cytotoxic T lymphocytes (CTLs) and effector CD4^+^ T cells	[62, 63]
Ginseng polysaccharides	Gastric cancer	Enhance the antitumor response to anti‐PD‐1 monoclonal antibody (anti‐PD‐1 mAb) by increasing gut microbiota‐derived metabolites, such as valerate	[64]

Abbreviations: HNSCC, Head and neck squamous cell carcinoma; SCFA, Short‐chain fatty acid.

However, the specific mechanisms through which these microbes interact with the tumor immune microenvironment, particularly their ability to induce the formation of TLS, are still to be explored in detail. In this paragraph, we summarize recent findings on the formation and role of TLS in infection‐associated cancers.

### 3.1 Microorganism‐Induced Cancers and TLS

It has been shown that the microbiota regulates local inflammation that plays a critical role in the formation of intratumoral TLS (Table 2) [8, 75]. For instance, the gut microbiota plays a key role in the formation and maturation of TLS, and directly influences TLS formation in tumors [66, 76].

**TABLE 2:** Correlations between microorganisms, cancers, and TLS formation.

Microorganism	Cancer type	TLS correlation (clarified)	Prognostic value	Mechanism of action	References
*H. hepaticus*	Colon (adjacent tumors)	*H. hepaticus* induces TpH cells promoting TLS maturation near tumors	Promotes maturation	Bacterial induction of Tfh cells leads to TLS adjacent to tumor	[66]
*E. coli*	Bladder cancer (MIBC)	*E. coli* stimulates Tfh and B‐cell responses, enhancing TLS formation and predicting PFS	Improved with PFS	*E. coli* antigens stimulate Tfh/B‐cell responses that structure TLS and enhance therapy outcome	[67]
MCPyV	Merkel cell carcinoma	No correlation between MCPyV infection and TLS presence	Independent	Unclear; TLS and MCPyV act as independent prognostic markers	[68]
EBV	Gastric cancer	TLS are prognostic only in EBV‐negative cases, not directly linked to EBV	Independent (in EBV^−^ only)	EBV presence not directly inducing TLS; other immune factors involved	[69]
EBV	Nasopharyngeal carcinoma	PD‐1^+^ CXCR5^−^ cells in TLS improve prognosis in EBV^+^ NPC	Improved	PD‐1^+^ CXCR5^−^ cells within TLS enhance antitumor responses	[60]
EBV	Gastroesophageal adenocarcinoma	CD8^+^ PD‐L1^+^ T cells in tumor center suggest immune regulation in EBV^+^ GOA	Context‐specific	Localization of immune cells suggests central tumor immune engagement	[70]
HPV	Cervical cancer	HPV infection significantly increases TLS and correlates with better prognosis	Improved	HPV drives TLS formation through viral antigens	[71]
HPV	HNSCC	HPV‐specific immune cells promote TLS formation and GC responses	Improved through GC response	HPV‐specific immunocytes initiate GC and TLS formation	[29, 72]
HV	Hepatocellular carcinoma	Increased TLS with CD8^+^ PD‐1^+^ T cells indicates CTL exhaustion in hepatitis‐infected HCC	Associated with CTL failure	Chronic infection increases immune cell infiltration leading to TLS with dysfunctional T cells	[73]
HBV	Intrahepatic cholangiocarcinoma	HBV infection supports TLS presence and reactivation of antitumor immunity	Improved via immune reactivation	HBV suppresses myeloid inflammation and reactivates immune surveillance via TLS	[74]

Abbreviations: *E. coli* , *Escherichia coli* ; EBV, Epstein–Barr virus; *H. hepaticus* , *Helicobacter hepaticus* ; HBV, hepatitis B virus; HPV, human papilloma virus; HV, Hepatitis virus; MCPyV, Polyomavirus with Merkel cells.

In these tissues, bacteria release pathogen‐associated molecular patterns (PAMPs), which activate signaling pathways such as Mitogen‐activated protein kinases (MAPK), Janus kinase/signal transducers and activators of transcription (JAK–STAT), and nuclear factor‐kappa B (NF‐κB). These pathways stimulate the secretion of cytokines and chemokines that promote TLS maturation (Figure 3). For example, colonization by *Helicobacter hepaticus* induces specific Tfh cells, promoting TLS formation in colorectal tumors [66]. Similarly, enrichment with *Lachnoclostridium* is associated with the presence of intratumoral TLS in hepatocellular carcinoma [76]. Likewise, *Salmonella*‐specific resident macrophages (CXCL13^+^ CX3CR1^hi^) induce TLS formation in situ [77]. The probiotic *E. coli strain Nissle* 1917 delivers cyclic di‐AMP (an agonist of cyclic guanosine monophosphate‐adenosine monophosphate synthase‐stimulator of interferon genes, cGAS‐STING), promoting DC‐mediated type I interferon production, which is crucial for TLS formation [78]. Innate immune activation by *Salmonella Typhimurium* leads to the release of pro‐inflammatory cytokines (i.e., IL‐1β, TNF‐α, and IFN‐γ), converting “cold” tumors into “hot” tumors and promoting TLS development [79]. In muscle‐invasive bladder cancer (MIBC), *E. coli* ‐specific Tfh and IgG directed against urothelium‐invasive *E. coli* predicted clinical benefit to pembrolizumab (an anti‐PD‐1 mAb) treatment. This therapy induced or upregulated TLS formation and improved progression‐free survival (PFS) [67].

Regarding viral infections (Table 2), studies in Merkel cell carcinoma (MCC) have shown that the presence of TLS is not correlated with Merkel cell polyomavirus (MCPyV) infection. Although patients with high expression of CXCL13 or CCL5, two chemokines strongly involved in TLS neogenesis, have a significantly better prognosis than those with low expression [80]. Similarly, in Epstein–Barr virus (EBV)‐associated gastric cancer, tumor‐infiltrating lymphocytes (TILs) and TLS appeared to be independent prognostic factors only in EBV‐negative cancers [81]. However, in EBV‐associated nasopharyngeal carcinoma (NPC), the presence of PD‐1^+^ CXCR5^−^ CD4^+^ Th‐CXCL13 cells within TLS may be associated with improved prognosis [43, 60]. In EBV‐positive gastroesophageal adenocarcinomas (GEA), about half of genome stable (GS) gastric cancers display TLS, while the majority of chromosomal instable (CIN) GEAs show T cell exclusion. In these CIN tumors, CD8^+^ T cells expressing PD‐L1 are found in the tumor invasive margin [70]. Moreover, human papillomavirus (HPV) infection, which is common in cervical cancer, has also been linked to increased TLS density and improved prognosis. In this study, TLS numbers correlated with the depth of tumor invasion, preoperative chemotherapy, HPV infection, and high levels of PD‐1, although there was no significant relationship between TLS and IL‐33, an alarmin for the immune system in the TME [71]. In HPV‐positive head and neck squamous cell carcinoma (HNSCC) patients, HPV‐specific immune cells appear to contribute to TLS formation and enhance survival through GC‐associated responses [29, 72, 82]. In HCC associated with chronic hepatitis virus infection, an enrichment of central‐memory CD8^+^ PD1^+^ T cells has been observed in TLS [83]. In intrahepatic cholangiocarcinoma, HBV infection and TLS presence have been linked to better prognosis in a subset of patients. This subgroup exhibited an enrichment of adaptive and innate cells, such as natural killer cells and activated DCs, likely limiting excessive myeloid inflammation and reactivating antitumor immunity [74].

Overall, while some discrepancies exist, most studies show that microorganisms induce the formation of mature TLS, which are associated with stronger immune responses and better prognoses in oncology.

### 3.2 Strategies to Induce Mature TLS Formation Through Microorganisms

If one agrees that microorganisms promote the formation of TLS and that TLS are associated with improved patient prognosis and enhanced responses to immune checkpoint inhibitors (ICIs), it becomes conceivable to leverage this relationship to induce TLS neogenesis through microbial engineering. Manipulating the composition of the bacterial microbiota or introducing engineered viruses or bacteria could therefore locally promote TLS formation, stimulate antitumor immune responses, and enhance treatment efficacy.

Several studies have explored this hypothesis. Notably, it has been demonstrated that the formation of mature TLS can be manipulated by targeting the gut microbiota, its metabolites, or associated signaling pathways [84]. This approach could pave the way for a new generation of cancer immunotherapies. For instance, intratumoral injection of *E. coli* MG1655 reprograms tumor‐associated macrophages toward a pro‐inflammatory phenotype producing CCL5, which enhances T‐cell infiltration and tumor clearance [85], suggesting that it can trigger TLS neogenesis.

Several cancer therapies have also shown to induce TLS formation [8, 86]. For instance, intramuscular vaccination targeting HPV16 E6/E7 oncoproteins in patients with cervical intraepithelial neoplasia (CIN2/3) successfully stimulated TLS development in the stroma close to intraepithelial lesions and increased CD8^+^ T cell infiltrate [87]. Similarly, in murine PDAC model, administration of antifibrotic a phosphates‐modified α‐mangostin nanoparticles containing a plasmid encoding the LIGHT protein (“nano‐sapper”) induced intratumoral TLS and inhibited abnormal collagen deposition [88]. Low‐dose radiotherapy (LDRT) combined with anti‐PD‐1 treatment also promoted the development and the maturation of TLS in a murine lung adenocarcinoma model. Interestingly, LDRT increased the formation of early TLS (defined by the authors as TLS lacking FDCs, and which represents an immature stage of TLS development) while the combination of LDRT with anti‐PD‐1 treatment led to TLS maturation. The enhanced antitumor effect observed with the combination was associated with increased CD8^+^ T cells within the TLS [89].

Modulating the microbiota composition through diet, fecal microbiota transplantation (FMT), or the use of probiotics, prebiotics, antibiotics, or even bacteriophages could further enhance TLS formation. In mouse models of colorectal cancer (CRC) and melanoma, oral gavage with commensal Clostridiales strains enhanced antitumor immunity by increasing infiltration and activation of intratumoral CD8^+^ T cells [90]. Many studies have shown that the diversity and composition of the intestinal microbiota are associated with the efficacy of immunotherapy and the incidence of immune‐related adverse events (irAEs). Commensal microbes can also activate CD8^+^ T cell‐dependent antitumor responses [85], and enhance antitumor immunity by activating DCs via Toll‐like receptor 4 (TLR4) signaling, particularly in murine melanoma models treated with radiotherapy, suggesting a DC‐mediated TLS neogenesis pathway [91]. Certain dietary interventions, such as the intake of fructo‐oligosaccharides (components of inulin fibers), can activate human macrophages to produce pro‐inflammatory cytokines involved in TLS formation [92]. In mice, an inulin‐rich diet can promote the production of microbiota‐derived bile acids that stimulate IL‐33 production [93], indirectly contributing to TLS formation, possibly via activation of IL‐33‐activated group 2 innate lymphoid cells (ILC2) [94]. Additionally, antibiotic therapy has been shown to alter the composition of the gut microbiota and its associated metabolites [95], suggesting that antibiotic therapy may indirectly impact TLS neogenesis and maintenance.

Intratumoral (i.t.) administration of inflammatory metabolites is another promising strategy. For example, the STING agonist ADU‐S100 injected i.t. into B16‐F10 melanoma has been shown to induce TLS formation and slow tumor growth [96]. This compound activated CD11c^+^ DCs, increasing the expression of TLS‐promoting factors (i.e., LTα, type I IFN, IL‐36) both in vitro and in vivo [96]. Activation of the cGAS–STING pathway also promoted the secretion of cytokines and chemokines (CXCL13, IFN‐γ) that support TLS establishment [97]. cGAS‐mediated TLS formation also enhanced humoral and antitumor responses in this setting. Moreover, increasing the number of Tfh by inducing their differentiation appears to be an effective strategy to trigger TLS formation in tumors [97]. Blocking immunosuppressive signaling pathways can also stimulate immune cell aggregation and facilitate TLS formation [86]. Additionally, depletion of specific regulatory cell populations, such as some macrophage subtypes or regulatory B cells (Bregs), may also promote TLS maturation in the tumor microenvironment.

## 4 Circulating Soluble Biomarkers of TLS in Cancers

Over the last two decades, an accelerating number of clinical studies have provided valuable insights into the potential role of TLS in overall survival (OS) and disease‐free survival (DFS) of patients with solid tumors, and in their response to immune checkpoint blockade (ICB) [7, 17, 18, 19, 98, 99]. These studies have highlighted the potential of TLS to guide clinical decision‐making and treatments. However, studying TLS in large cohorts of patients remains limited primarily due to challenges with its detection [99]. Currently, TLS identification relies almost exclusively on techniques such as immunohistochemistry (IHC) and immunofluorescence (IF), performed on histopathological slides obtained from tissue biopsies or surgical specimens [100]. These techniques require not only access to fresh or well‐preserved tissue samples, but also technical expertise to combine multiple specific cellular markers, typically T cell markers (e.g., CD3), B cell markers (e.g., CD20), FDC (e.g., CD21, CD23), and HEV‐type endothelial markers (e.g., PNAd). Furthermore, the interpretation of these stains, which is essential to confirm structured lymphoid organization, remains subject to interobserver variability and requires standardization [101]. Additionally, these data are collected postsurgery, that is, after tumor resection. This reliance on invasive, technically demanding tissue analyses significantly limits the large‐scale evaluation of TLS in patient cohorts and clinical contexts where pathological tissue is often inaccessible or unavailable. Moreover, it does not allow for the early identification of patients likely to respond to ICB treatments prior to complex surgical interventions.

**FIGURE 4:**
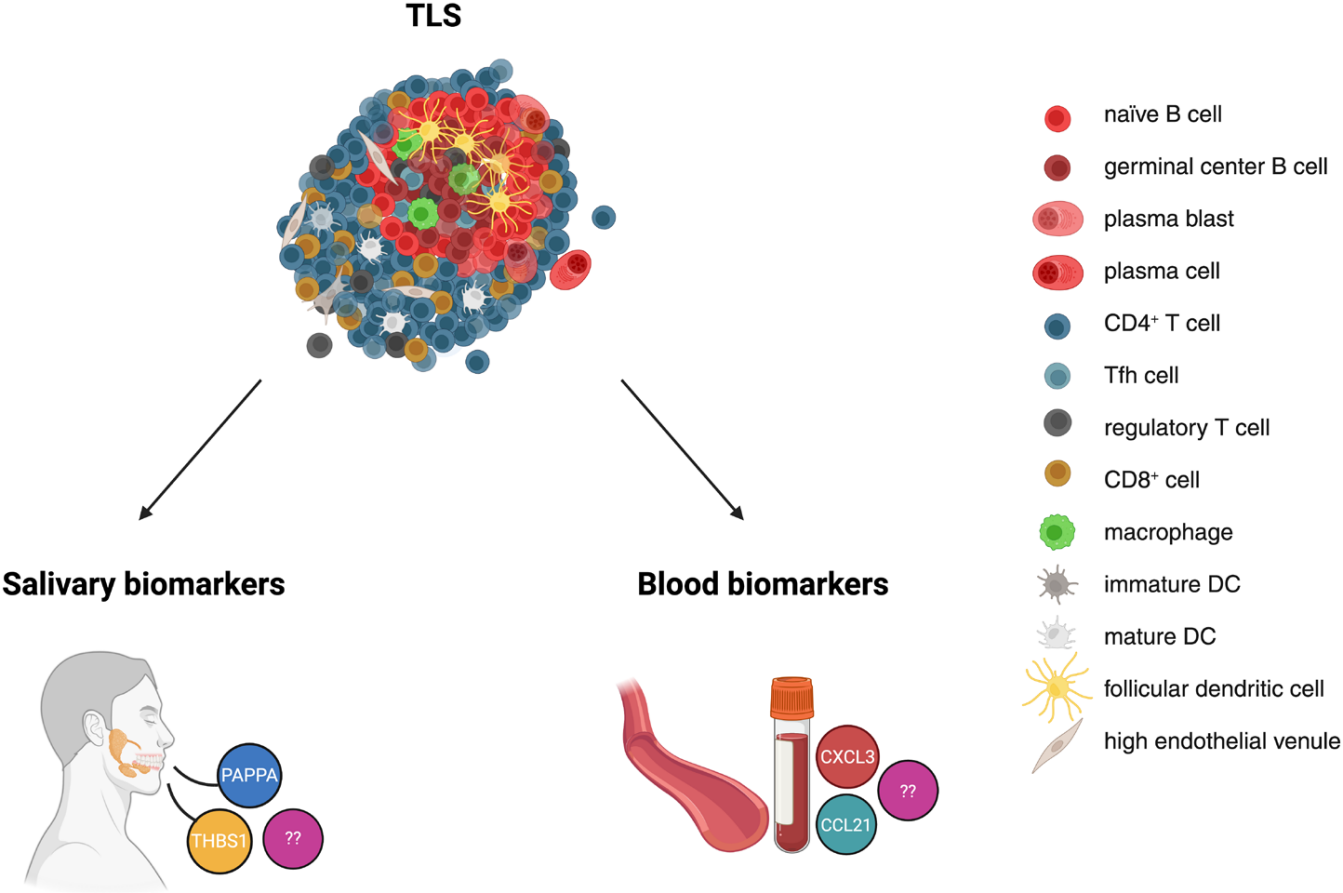
Identifying TLS‐specific molecular signatures in body fluids. Created in https://BioRender.com.

Identifying TLS‐specific molecular signatures in body fluids could therefore offer faster, less invasive diagnostic approaches (Figure 4). Some studies in malignant and autoimmune diseases have proposed candidate molecules as potential indicators of TLS or GC presence. For instance, salivary biomarkers indicative of ectopic GCs in the salivary glands of patients with Sjögren's syndrome (an autoimmune disorder marked by chronic inflammation of exocrine glands, particularly the salivary and lacrimal glands) have been investigated using a multiplex immunoassay targeting 187 proteins [102]. Two biomarkers (Table 3), the Pregnancy‐Associated Plasma Protein A (PAPP‐A) and the Thrombospondin‐1 (THBS1), not found in simple hyposialia, predicted the presence or absence of GCs with 93.8% accuracy. These proteins are involved in processes such as macrophage chemotaxis, apoptotic cell clearance, and alterations in growth factor signaling. Unlike simple glandular inflammation (measured by focus score), TLS presence induced the most pronounced changes in the salivary proteome [102]. Another study performed in patients with primary Sjögren's syndrome (pSS) [107] has proposed that CXCL13, a chemokine produced by CD4^+^ Tfh cells and FDCs, represents a serum biomarker for the presence of ectopic GCs in the minor salivary glands of pSS patients (Table 3). Salivary CXCL13 levels were also elevated in GC^+^ patients, but with a lower discriminative power [107].

**TABLE 3:** Blood and salivary circulating biomarkers of TLS.

Biomarker	Biological fluid	Associated pathologies	TLS relevance	Prognostic value	References
PAPPA	Saliva	Primary Sjögren's syndrome	Associated with ectopic germinal centers in salivary glands	93.8% accuracy in GC prediction	[47]
THBS1	Saliva	Primary Sjögren's syndrome	Same as above	93.8% accuracy in GC prediction	[47]
CXCL13	Serum/Saliva	Sjögren's syndrome, ovarian cancer, colorectal cancer	Strongly expressed in mature TLS; reflects TLS and GC activity	Correlates with disease severity, therapeutic response, and survival	[49, 103, 104]
CCL21	Serum	NSCLC, Primary Sjögren's syndrome	Correlated with TLS maturity, immune activation, and response to immunotherapy	Potential predictive marker of immunotherapy efficacy	[44, 45, 50, 105, 106]

Abbreviations: NSCLC, Nonsmall cell lung cancer; PAPP‐A, Pregnancy‐Associated Plasma Protein A; THBS1, Thrombospondin‐1.

A recent study demonstrated TLS presence in the lacrimal glands (LGs) of NOD and NOR mice, which are classical models of Sjögren's syndrome [108]. The authors established a functional link between TLS in the LGs and the local production of IgA and IgG autoantibodies in tears, distinct from those found in serum. A comparative analysis of autoantibody profiles revealed fluid‐specific signatures with certain autoantibodies (i.e., anti‐Mi‐2, Jo‐1, TPO, PL‐7, SAE1/SAE2) found exclusively in tears but not in blood, and a predominance of the IgA isotype. Single‐cell RNA sequencing (scRNA‐seq) analysis of lacrimal glands confirmed the presence of plasma cells expressing *Igha* and *Ighg2b* transcripts, demonstrating their role in local antibody production [108].

Similarly, in patients with colorectal cancer receiving first‐line oxaliplatin‐based therapy [109], serum CXCL13 was identified as a promising biomarker of TLS formation (Table 3). CXCL13 levels increased significantly in responders after 12 weeks of treatment and decreased in nonresponders. This increase correlated with improved OS and PFS. Transcriptomic analyses and in silico data further showed that high tumor CXCL13 expression was associated with higher B cell and CD8^+^ T cell infiltration and a TLS‐related gene signature. These findings suggest that blood CXCL13 reflects the presence of active intratumoral TLS and could serve as a noninvasive marker of therapeutic response and favorable prognosis [109]. Serum CCL21 may also represent a relevant biomarker for TLS presence, although available data remain limited. In NSCLC, one study showed that patients with a history of smoking exhibited elevated serum CCL21 levels, which were associated with increased intratumoral TLS density and maturity, as well as improved responses to immunotherapy [110].

In conclusion, among all the molecules studied (Figure 4 and Table 3), CXCL13 appears to be the most extensively documented circulating biomarker associated with TLS or GCs presence in both serum and saliva. Other cytokines and chemokines, although critical for TLS formation, have mostly been studied in tissues, and data on their detection in biological fluids remain limited in the context of TLS. Clearly, the analysis of soluble biomarkers locally associated with TLS formation (for instance, saliva in oral cancers or uterine and vaginal secretions in cervical or ovarian cancers) is an emerging and promising field.

## 5 Lymphoid Structures and Peripheral Nervous System in Cancer

The formation of lymphoid aggregates and TLS in solid tumors is strongly dependent on the inflamed tumor microenvironment (TME), where Lymphoid Tissue‐inducer (LTi)‐like cells and Lymphoid Tissue‐organizer (LTo, of stromal origin) cells play a central role in recruiting naïve and memory lymphoid cells via mature HEVs. Remarkably, it is now well documented that the TME interacts with the peripheral nervous system (PNS) through a network of sensory neurons and sympathetic nerves present in tumors, which can dialog with both innate and adaptive immune cells [111, 112, 113, 114, 115]. Accordingly, the potential cross‐talk between the PNS and TLS in the TME is only beginning to be explored. The relevance of this investigation is strongly supported by studies on the presence and role of nerve fibers in SLO, mainly spleen and LNs, which contain both sensory and sympathetic neurons [116, 117, 118].

### 5.1 Sensory Neurons as Modulators of Immune Organizations

Sensory (part of nociceptor) neurons innervate the spleen along blood vessels and are found in B‐cell zones where they promote GC formation and antibody response [119]. In LNs, these neurons are primarily peptidergic nociceptors, as demonstrated in studies of mouse popliteal LNs [120]. More than 95% of sensory neurons were found to express Nav1.8, with the majority (> 80%) also expressing the tropomyosin receptor tyrosine kinase A (TrkA, the high‐affinity receptor for nerve growth factor [NGF]) and the calcitonin gene‐related peptide (CGRPα, encoded by the *Calca* gene). In this study, sympathetic neurons, characterized by their expression of tyrosine hydroxylase (TH) and production of catecholamines, which are neurotransmitters capable of interacting with immune cells, were also detected in popliteal LNs [120].

Both sensory and sympathetic neurons form a complex network of nerve fibers that can dialog with immune cells present in LNs. However, they may harbor different functions in their cross‐talks with immune cells. For instance, differences in their localization in mouse popliteal LNs have been emphasized by von Andrian and colleagues [120]: (i) sensory neurons branch extensively into the LNs interstitial space, whereas TH^+^ sympathetic nerve fibers remain mostly around blood vessels, even though the initial entry paths of both types of nerves into LNs closely follow blood vessels in the *hilus* region [118]; (ii) sensory neurons are preferentially found at the periphery of LNs and are scarcely present in the deep cortex where most naïve lymphocytes reside, suggesting that TH^+^ sympathetic nerve fibers are more prone to interact directly with these cells than their sensory counterparts; (iii) sensory fibers are almost entirely absent from postcapillary peripheral node addressin (PNAd)^+^ HEVs, a hallmark of mature TLS, through which naïve and memory B and T lymphocytes migrate into TLS, as observed in LNs [42, 121, 122].

Thus, similar to LNs, a cross‐talk between TLS and nerve fibers, both sensory and sympathetic, may occur at different stages of TLS formation and maintenance. Sensory innervation often increases at sites of inflammation, suggesting that sensory neuronal networks may undergo remodeling in response to inflammatory signals, which are also critical for TLS neogenesis [8]. However, the role of sensory neurons in tissue inflammation remains complex, as both pro‐ and anti‐inflammatory activities have been reported. On the one hand, nociceptor neurons can mitigate inflammatory responses to bacterial infections [123], while on the other, the presence of cutaneous nociceptor neurons is required for the production of the pro‐inflammatory interleukin (IL)‐23 by dermal DCs [124]. Thus, TLS and surrounding inflamed tissues are likely to dialog positively or negatively with local sensory neurons during different stages of their neogenesis. This potential interaction was examined in cancer using a mouse melanoma model [125]. Sensory neurons were ablated either chemically (by intraperitoneal, i.p., injection of resiniferatoxin, which selectively ablates transient receptor potential cation channel, subfamily V, member 1 (TRPV1)^+^ sensory neurons), or surgically, through denervation of dorsal thoracic sensory cutaneous fibers, preventing axon sprouting and reinnervation, and resulting in complete loss of nociception of the back skin. The authors demonstrated that the absence of sensory neurons (i) moderately slowed the growth of subcutaneously injected B16‐F10 melanoma cells and (ii) was associated with an increased intratumoral expression of genes encoding PNAd, CD34, ICAM‐1, VCAM‐1, and CCR7, all markers of HEVs. Consistent with these RNA‐based analyses, increased PNAd labeling of HEVs was observed in tumors from denervated mice, indicating that sensory neuron loss promotes HEV formation and maturation. Moreover, sensory nerve ablation led to upregulation of 54 genes previously related to TLS formation [125]. Importantly, this included genes encoding known key mediators of TLS neogenesis, such as the lymphotoxin receptor (LTβR) agonists LIGHT, LTα, LTβ, and TLS/HEV‐related chemokines including CXCL9, CXCL13, CCL3, CCL5, CCL19, CCL21, and the pro‐inflammatory cytokine TNF‐α. Taken together, these data suggest that sensory neurons contribute to tumor progression and negatively regulate TLS formation in subcutaneous melanoma, although the molecular machinery underlying this inhibition remains to be elucidated. In addition, sensory nerve ablation resulted in (i) denser B‐cell and T‐cell aggregates adjacent to HEVs, and (ii) larger T lymphocyte clusters surrounding tumor‐associated blood vessels. An increase in the B and T cell repertoires of tumor‐infiltrating lymphocytes was also observed in denervated mice. However, GCs were absent in the B‐cell aggregates from these animals, despite an increase in B cell numbers around mature HEVs. This suggests that these tumor‐infiltrating B cells may have originated from SLO [125]. This hypothesis was reinforced by the fact that, astonishingly, no GCs were detected in the TME of denervated mice at any time points after B16‐F10 implantation, indicating that the final stage of TLS maturation is not reached—or is markedly delayed—in the absence of sensory neurons. Supporting this further, in a murine infection model, treatment of nondenervated animals with a depleting anti‐CD8 antibody prior to lung infection with *Staphylococcus aureus* resulted in reduced B‐cell infiltration and absence of GCs [126]. Similarly, in a melanoma model, anti‐CD8^+^ treatment of denervated mice before B16‐F10 subcutaneous (s.c.) injection led to a marked decrease in peri‐HEV B cells [125]. This raises the question of whether the negative influence of skin sensory nerves on TLS formation and antitumor immune responses in this preclinical model also applies to melanoma patients. Analyses of skin cutaneous melanoma (SKCM) RNA‐seq data from The Cancer Genome Atlas (TCGA) revealed that patients with low summed expression of genes associated with sensory neurons, such as *Calca*, *Gal*, *Kcnn1*, *Kcnh2*, *Nefh*, *Scn10a*, and *Tac1* (expression specifically observed in both murine and human sensory neurons) exhibited an upregulation of HEV maturation and immune response pathways. Interestingly, these pathways included leukocyte diapedesis and LTβR signaling. Remarkably, the overall survival was significantly higher among melanoma patients with low Schwann cell (SC) mRNA content [125]. All these data strongly suggest that reduced sensory innervation within the TME promotes TLS neogenesis and enhances antitumor responses in mouse models and possibly human melanoma.

### 5.2 Sympathetic Neurons as Modulators of Immune Organizations

Sympathetic nerves could also influence the formation, maintenance, and function of TLS, possibly independently of sensory neurons, as demonstrated in SLOs. Indeed, it has been shown that LNs receive mutually independent sympathetic and sensory innervation using selective ablation of each type of nerve fibers [120]. Ablation of Nav1.8^+^ neurons in Nav1.8Cre/+3Rosa26DTA/tdTomato (Nav1.8‐DTA) mice [127] resulted in the loss of sensory fibers without affecting TH^+^ sympathetic fibers. Conversely, ablation of TH^+^ sympathetic fibers using 6‐hydroxydopamine (6‐OHDA), a selective catecholaminergic neurotoxin, had no impact on Nav1.8^+^ sensory fibers. This demonstrated that each type of innervation does not depend on the presence of the other. Thus, what can be taught from the sympathetic nerves/LNs dialog concerning the control of TLS neogenesis? In a mouse study, surgical transection of the sciatic nerve, which consists predominantly of sensory (about 70%) and sympathetic (about 25%) fibers, with only 5% motor fibers [128, 129], led to significant popLN expansion. This was attributed to (IFN)‐γ production by popliteal LNs CD8^+^ T cells, leading to a massive recruitment of leukocytes, predominantly T cells, through an IFN‐γ‐dependent upregulation of ICAM‐1 on HEVs [130]. Importantly, treatment of denervated animals with the β2‐adrenergic receptor (Adrb2) agonist clenbuterol reversed the denervation phenotype, reducing both the number of popliteal LNs CD8^+^ T cells and their intracellular levels of IFN‐γ. This demonstrated that Adrb2‐expressing cells are central mediators of sympathetic nerve signaling in popliteal LNs. Of note, 6‐OHDA treatment also increased IFN‐γ expression, although the study did not report whether cellularity was similarly affected in this setting [130]. Based on these data, the authors suggested that sympathetic signaling participates in the inhibition of LN overreaction to immune challenge and exerts an anti‐inflammatory role, which is lost upon sympathetic denervation. However, it is well established that patients with nerve injury, including sympathetic nerves, often exhibit reduced immunity [131]. Therefore, the effect of sympathetic nerves on lymphoid organs could be driven by the level and/or nature of the inflammatory context, as well as the tissue microenvironment, either hindering or favoring immune responses.

These observations of sympathetic nervous system (SNS) cross‐talk with LNs have led to investigate how sympathetic nerves may influence TLS formation and function, and whether they play positive or negative roles in the elaboration of immune responses under immunogenic challenge. It has been reported that 6‐OHDA‐mediated denervation of sympathetic neurons induces the collapse of arterial plaque‐associated lymphoid structures [132]. By contrast, other authors showed that selective surgical ablation of the parasympathetic *vagus* nerve—but not that of sympathetic nerve fibers—impaired TLS formation in dextran‐sulfate sodium (DSS)‐inflamed colon. This was associated with a marked decrease in CXCL13 expression by podoplanin (Gp38^+^)‐expressing stromal cells. Surprisingly, these authors found that TLS neogenesis in DSS‐inflamed colon was independent of the LTα1β2/LTβR axis, as shown using LTα^−/−^ mice, although no FDCs were observed in the lymphoid aggregates of these mice [133]. Our laboratory has also examined the impact of chemically induced sympathetic denervation on TLS by developing a mouse model in which immunocompetent mice received 6‐OHDA treatment before intranasal instillation of lipopolysaccharide (LPS), an inducer of pulmonary TLS in this setting [11]. TLS quantification was performed on the whole lung volume by hematoxylin and eosin counterstaining and analysis of scanned single‐plane images using artificial intelligence (AI) software trained to recognize all types of lymphoid aggregates. These results showed a significant decrease in both the number and surface areas of TLS in the lungs of denervated mice. This reduction was not due to a decreased lymphocyte recruitment or lung inflammation provoked by the reduction of pulmonary alveolar space after 6‐OHDA treatment and, hence, was not due to a reduced inhalation of LPS. The expression of *Tnfa*, *Il1b*, and *Ifng* induced by LPS remained as elevated in 6‐OHDA‐treated animals as in innervated LPS‐instilled animals. Thus, the absence of local sympathetic nerve fibers may result in a shortage of molecules involved in the interaction between lymphoid tissue‐inducer (LTi)‐like cells and stromal lymphoid tissue‐organizer (LTo) cells during the early stages of TLS formation within inflamed lung tissue. This possibility remains to be investigated, especially knowing that the study did not account for a potential role of sensory neurons in TLS formation following LPS instillation. This is an important issue since part of these neurons may express TLR4 (CD284), a LPS coreceptor, as suggested by a gene expression study performed in LNs [120]. An alternative hypothesis is that the absence of sympathetic neurons affects the B‐ and/or T‐cell compartments, thereby impairing TLS formation and maintenance. Remarkably, after LPS intranasal instillation, a significant drop in CD23^+^ naïve cells among total B cells was observed in the lungs of 6‐OHDA‐treated animals compared to innervated mice. However, no such decrease in naïve B cells was seen in the spleen of these denervated mice [11]. It suggests that sympathetic nerve fibers contribute to the presence of a naïve B cell pool in inflamed lungs, hampering both TLS formation and, likely, the local antibody primary response. Remarkably, B cells express β2‐adrenergic receptors [134] as well as dopamine receptors [135]. In addition, follicular Tfh cells produce dopamine upon cognate interaction with B cells, a process that enhances the Tfh‐B cell synapse areas and GC productive synapses [136]. Thus, sympathetic nerve fibers may positively regulate the antibody response elaborated within TLS by acting on the B cell compartment and promoting GC formation through the local release of catecholamines. Interestingly, it has been reported that IgM primary antibody response to bovine serum albumin (BSA) and peripheral blood B cell number are decreased in rats producing only minute amounts of noradrenaline (norepinephrine), a major neurotransmitter released by sympathetic nerve fibers in the spleen and LNs [137]. In this study, low noradrenaline level was achieved by silencing the rat glutaminase (GLS) gene in glutamatergic neurons of the cerebellar interposed nucleus (IN). This led to a strong decrease in norepinephrine content in the spleen and mesenteric LNs [137]. This effect likely occurred via cerebellar IN glutamatergic projections to the hypothalamus, an organ known to exert its immune regulation through sympathetic nerves in lymphoid tissues [73]. Thus, we hypothesize that sympathetic nerves participate in the control of TLS formation and maturation in inflamed tissues, including cancer, through the local release of neurotransmitters such as dopamine (also produced by Tfh cells within TLS), adrenaline (epinephrine), and noradrenaline (norepinephrine), which act on the B cell compartment via the dopamine and/or β2‐adrenergic receptors. Indeed, nerve fibers have been observed in close proximity to B‐cell areas within lymphoid aggregates in inflamed cancer tissues. In pancreatic ductal adenocarcinoma (PDAC) patients, B cell‐containing lymphoid aggregates have been detected in the close vicinity of PGP9.5^+^ (a pan‐neuronal marker) neurons, although the type of neurons was not defined, and it remained unclear whether these aggregates represent bona fide TLS [138]. Additionally, it has been reported that doublecortin (DCX^+^) neural progenitors from the central nervous system (CNS) can infiltrate prostate tumors and differentiate into new adrenergic sympathetic neurons [139]. In this study, based on the Hi‐Myc prostate adenocarcinoma mouse model, the genetic depletion of DCX^+^ cells, as well as the surgical or chemical ablation of sympathetic nerves, inhibited prostate tumor growth and metastases. This suggests a protumoral role for sympathetic‐derived neurotransmitters. However, the role of immune cells in this setting has not yet been investigated.

### 5.3 Neurons and TLS: A Dialog Still to Decipher

Overall, the study of PNS–TLS interactions in cancer is still in its infancy, and only a limited number of publications have addressed this issue to date. One report indicated that sensory nerves hinder the formation of TLS in subcutaneous melanoma tumors [125]. However, sensory neurons can exhibit pro‐ or anti‐inflammatory activity depending on the tissue being studied [123, 124]. This opposite effect on inflammation may, therefore, positively or negatively impact TLS formation and maturation, particularly in the context of cancer. The role of sympathetic nerves in TLS formation is also unclear. Some reports suggest that the presence of these nerves is needed for TLS neogenesis and maintenance [11, 132], possibly due to the local release of catecholamines that can act on the B cell compartment via dopamine and/or β2‐adrenergic receptors expressed by these cells. However, another study showed that depletion of sympathetic neurons does not inhibit the formation of TLS in DSS‐inflamed colon [133]. Ongoing studies in preclinical models and cancer patients are expected to provide a better understanding of the SNS–TLS relationship in different tumor contexts, as well as the underlying cellular and molecular mechanisms.

## 6 Humanized Mice: A Reliable Tool for Human TLS Studies?

Although the study of various clinical situations in patients has proven to be very useful to get insights into the relationship between TLS and tumors, the use of animal models has proven to be very helpful in deciphering the underlying cellular and molecular mechanisms involved in TLS neogenesis, and humanized mice hold significant promise. Moreover, the cascade of events and molecular interactions occurring between cells from different tissues (lymphoid, nervous, vascular, connective, etc.) that lead to TLS formation remains incompletely understood. This question is of primary importance to fully evaluate the possibility of inducing TLS in cancer patients who otherwise lack these beneficial structures. Several therapeutic strategies aimed at inducing TLS have been recently tested in preclinical mouse models [8], and new molecular targets are actively being investigated for this purpose. However, syngeneic mouse tumor models may fail to fully recapitulate the complex TLS architecture observed in cancer patients, which argues in favor of studying human biopsies and tumor samples. But again, mechanistic studies in humans are difficult to conduct, and transposition of results obtained from murine models to humans should be done with caution. Although in vitro bioengineering approaches have been recently proposed to generate human LN‐like organizations and TLS [140, 141, 142], these approaches still face formidable challenges due to the complexity of in vivo formation and maturation of TLS. In vivo generation of human TLS in autoimmune or cancer models may enable mechanistic studies and yield results more relevant to clinical settings. Humanized mice that are immunodeficient animals (such as NSG or nonobese diabetic (NOD)‐scidIL2rgnull mice) reconstituted with CD34^+^ human hematopoietic progenitors (CD34‐HPs) represent a promising tool for this purpose. We will therefore review the current evidence for the presence of TLS in such humanized mice. Of note, we will not address results obtained from humanized mouse models based on the transfer of human peripheral blood lymphocytes (PBLs). Indeed, these models invariably lead to T cell expansion and progressive graft‐versus‐host disease (GVHD), with a fatal outcome. Although HLA‐transgenic mice exhibit a less severe form of GVHD [143], the lack of B cells and myeloid cells after the transfer of PBLs makes this model unsuitable for TLS research.

### 6.1 Of TLS in Autoimmune Diseases in Humanized Mice

TLS have been first reported in patients with autoimmune disease. Thus, their presence would be expected in autoimmune models using humanized mice. However, such models are quite rare in CD34^+^‐reconstituted mice, since autoimmunity is rarely determined at the level of CD34^+^‐HP, but rather depends on environmental factors and/or tissue degeneration. A notable exception to this rule is the transfer of CD34^+^‐HPs deficient in the *Forkhead box P3* (*FOXP3*) gene, which results in intense autoimmune syndromes in humanized mice, mimicking the Immunodysregulation polyendocrinopathy enteropathy X‐linked (IPEX) disease [144]. Unfortunately, the presence of TLS cannot be inferred from the pictures provided in that study. Likewise, targeting regulatory T cells (Tregs) in CD34^+^‐humanized mice using the antihuman cytotoxic T‐lymphocyte‐associated protein 4 (CTLA‐4) monoclonal antibody (mAb) ipilimumab leads to lymphoproliferation and liver inflammation, resembling responses seen in treated patients [145]. Some clusters of human immune cells were observed in the liver of treated mice, but whether these constitute *bona fide* TLS remains to be established. Another avenue for studying autoimmunity in humanized mouse models is to inject or vaccinate CD34^+^‐reconstituted mice with chemicals or compounds capable of initiating inflammatory responses that lead to autoimmune syndromes. One example is the injection of complete Freund's adjuvant (CFA) in the knee joint of humanized NSG mice to mimic arthritis [146]. Although the search for TLS was not a primary objective of that study, aggregates of human CD45^+^ cells were observed in the joints of the animals and disappeared after anti‐TNFα treatment. Thus, the presence of TLS in CD34^+^‐humanized mouse models of autoimmunity is still a matter of debate and should be analyzed more systematically. Most autoimmune models in humanized mice often involve transferring T cells from patients with autoimmune diseases, but B cells are typically missing, which may prevent the formation of TLS in these models.

### 6.2 Of TLS in Immuno‐Oncology in Humanized Mice

In contrast to autoimmunity, CD34^+^‐humanized mice are a valuable tool for immuno‐oncology (IO) research [147]. The simultaneous presence of human tumors and human immune cells in the same animal offers a formidable opportunity for studying fundamental mechanisms underlying tumor/immunity cross‐talk as well as delving into promising therapeutic strategies with drugs designed for human usage. For instance, CD34^+^‐humanized mice have been used to show improved efficacy of an anti‐ICOS mAb when combined with chemotherapy [148]. Within the IO framework, humanized mouse models could be employed to gain insights into the rules of TLS formation according to the nature of the tumor and/or its associated microenvironment. Establishing these rules may pave the way for novel TLS‐inducing therapies with higher translational potential to humans compared to murine models. However, the major limitation of humanized mouse models remains the allogenic nature of the antitumor T‐cell response, as opposed to the HLA‐restricted autologous response directed to tumor antigens that takes place in patients. It should be stressed that allogenicity does not obliterate the relevance of these models since T‐cell activation and function remain dependent on TCR‐MHC class I and class II interactions [149]. Another limitation is the lack of SLO in these models, which impairs B‐cell response. Only recently humanized mice with LNs and functional germinal centers following immunization, have been described. Concerning the presence of TLS in humanized tumor‐bearing mice, it has been shown that TLS‐like structures can be observed upon immune checkpoint blockade (ICB) treatment. The first example of this is the transplantation of patient‐derived tumoroids derived from patients with microsatellite instability‐high (MSI‐H) CRC in the cecum of humanized mice [150]. These mice developed primary tumors along with liver and peritoneal metastases after organoid transplant. Treatment with anti‐CTLA‐4 (ipililumab) or PD‐1 (nivolumab) mAb led to complete clearance of liver metastases and a reduction in primary tumor size, although peritoneal metastases remained unaffected. Remarkably, a large number of TLS‐like aggregates containing antigen‐presenting‐competent B cells was observed after ICB treatment in the liver. These structures exhibited a central zone of CD20^+^ B cells surrounded by CD8^+^ T cells. These structures were also found at the periphery of some primary tumors, but to a significantly lower extent in humanized mice. They were not detected within or in the close vicinity of peritoneal metastases. Notably, the number of tumor‐infiltrating human CD45^+^ leukocytes increased in primary cecum tumors, and liver and peritoneal metastases. However, ICB treatment did not affect the infiltration of FOXP3^+^ Tregs and CD68^+^ macrophages at any tumor sites (primary tumor and metastases). Strikingly, ICB treatment triggered B cell infiltration into primary cecum tumors and liver metastases, but not into ICB‐refractory peritoneal metastases. Interestingly, these peritoneal metastases were characterized by high levels of immunosuppressive cytokines (e.g., IL‐10, TGF‐β1, TGF‐β2, TGF‐β3) in ascitic fluid. Finally, treatment with an anti‐CD20 mAb (rituximab) depleted B cells from all tumor sites (primary tumors and metastases) and abrogated the ability of anti‐CTLA‐4 therapy to clear liver metastases [150]. Collectively, all these data suggest that (i) lymphoid aggregates can develop in humanized mice bearing human tumors and treated with ICB, and (ii) humanized mice make it possible to perform in‐depth analyses of B cell role in controlling primary tumors and metastatic tumors, potentially in conjunction with these lymphoid aggregates. However, although these lymphoid aggregates were referred as TLS‐like in this study, several key markers essential for definitively defining TLS, such as the HEV‐specific peripheral node addressin (PNAd) marker or CD21^+^ CD23^+^ FDCs, were not detected in this study.

To our knowledge, the most compelling evidence for the presence of TLS‐like in humanized mice has been shown in a model of localized and disseminated gastrointestinal stromal tumor (GIST) [151]. In this study, tumor‐bearing humanized mice were treated with a fusion molecule that contains a cancer vascular targeting peptide (VTP) that “normalizes” GIST angiogenic blood vessels, and a TNF superfamily member, LIGHT (TNFS14), a previously recognized TLS inducer in mice. GIST was either implanted subcutaneously, mimicking primary GIST in patients, or injected intraperitoneally to allow the dissemination of tumor cells. LIGHT‐VTP treatment of GIST in humanized mice improved vascular function and tumor oxygenation, as measured by quantifying intratumoral hypoxia‐probe deposits. This paralleled an increase in intratumoral human granzyme B (GrzB)^+^ effector T cells. Furthermore, intratumoral HEVs and lymphoid aggregates potentially corresponding to immature TLS and resembling spontaneous TLS observed in GIST patients, were also detected following LIGHT‐VTP treatment. These lymphoid aggregates contained loosely organized T and B cells, as well as HEVs, although no GCs were observed. The presence of CD21^+^ CD23^+^ FDCs was not assessed. Consequently, it remains unclear whether the lymphoid clusters induced by the LIGHT‐VTP treatment constitute true immature TLS. Therefore, current evidence does not support the formation of fully mature TLS in humanized mice engrafted with human tumors, as shown in these two studies [150, 151]. This challenges the notion that humanized mice are suitable for studying TLS neogenesis and function in a human immune context. This limitation is not unexpected, given the lack of a fully mature B cell compartment and the absence of SLO in standard humanized mouse models. Therefore, it is likely that TLS should be searched in models prone to LN development. There are two reports of “spontaneous” LN formation in the BALB/c Rag2^−/−^ Il2rg^−/−^ Sirpa^NOD^ (BRGST) [152] and THX [152] mice, which differ from the standard NOD.SCID.gamma‐c null mice in their genetic backgrounds. Bone marrow/Liver/Thymus (BLT) immunodeficient mice reconstituted with CD34^+^ HPs and grafted with fetal human tissues—will not be discussed here since they are not widely available [153]. BRGST mice are BALB/c mice crossed onto the RAG‐gamma‐c null background in which the signal‐regulatory protein‐α (SIRPα) allele from NOD mice was introduced to support human xenografts. Thymic stromal lymphopoeitin (TSLP) transgenic mice were back‐crossed onto the Balb/c Rag2^−/−^ Il2rg^−/−^ Sirpa^NOD^ strain (BRGS) to generate TSLP‐transgenic BRSG (BRGST) mice that overexpress TSLP, a cytokine that exhibits structural and functional homology to IL‐7. This strategy turned out to be extremely successful since those mice developed LNs (but no Peyer's patches). TSLP expression during embryogenesis rescued fetal LTi cell function in these mice, in which the IL‐7 signaling pathway is blocked due to the gamma‐c‐null mutation. Unfortunately, it was later discovered that these BRGST mice develop atopic dermatitis, characterized by severe skin injuries, elevated Th2 and Th22 cells, and increased serum IgA and IgE levels. Thus, the development of SLOs in BRGST humanized immune system (HIS) mice comes at the cost of a severe autoimmune syndrome. The reverse could also be true, as SLO could result from the inflammatory state associated with a Th2‐mediated immune response in these mice. Whether TLS can be generated in inflamed tissues in these mice is still an open question. In particular, evidence for tumor‐associated TLS in tumors implanted in humanized BRGST mice is still lacking and may be difficult to demonstrate in the context of concomitant autoimmune syndrome.

A second example of humanized mice with “spontaneous” LN development is the recently described THX mice [154]. These animals are derived from immunodeficient NSGW41 (NOD.Cg Kit^W41J^ Prkdc^scid^ Il2rg^tm1^Wjl/WaskJ) or NBSGW (NOD.CgKit^W41J^ Tyr^+^ Prkdc^scid^ Il2rg^tm1^ Wjl/ThomJ) mice in which immune reconstitution does not rely on myeloablation. Further improvement in the reconstitution of a human immune system is achieved by administration of 17β2‐estradiol in the drinking water *ad libitum* from 14 weeks of age onward. 17β2‐estradiol supports differentiation of human hematopoietic stem cells (HSCs), lymphoid and myeloid immune cells, since all these cells express estrogen receptors ERα and ERβ. This protocol is particularly effective, as it enables the detection of peripheral SLOs and Peyer's patches, following immunization. The ability of THX mice to generate TLS in inflamed tissues has not been examined in this setting. Remarkably, THX mice are able to mount fully mature neutralizing antibody responses against various pathogens. However, they also develop lupus‐like symptoms, including the production of auto‐antibodies when challenged with pristane. Of note, the study reported by Casali and colleagues does not indicate whether LNs were observed before immunization. A possible explanation for these different observations is that SLO formation occurs only as a consequence of immunization, due to the activation of the LTi‐like cell/LTo cell cross‐talk in adult animals.

In conclusion, published results support the hypothesis that humanized mice may represent a promising platform to study and decipher the mechanisms and functions of TLS during the immune responses to tumors. However, it is of utmost importance to investigate in detail the generation of LTi cells in humanized mice, as these cells are essential for the proper development of TLS. More research is needed to define the best conditions for TLS formation in a given type of cancer using humanized mouse models that are feasible in moderately equipped laboratories.

## 7 Concluding Remarks

It is now well established that TLS play a pivotal role in shaping antitumor immunity and are critical to clinical responses to immune checkpoint inhibitors. Over the last decade, the importance of TLS‐B cells as key players in the control of solid tumors has revolutionized the previously dominant view that only T cells are the major effectors of antitumor immunity. Although numerous studies have explored the structure and function of TLS, important issues remain to be addressed to enable their inclusion in our immunotherapeutic arsenal. In particular, numerous microorganisms and their metabolites have been shown to influence TLS neogenesis, offering new insights into the interplay between microbes, inflammation, and tumor progression. While some pathogens promote tumor growth, others enhance antitumor responses by stimulating TLS formation and improving patient prognosis. Harnessing microbial agents or modulating the microbiota thus represents a promising strategy to induce mature TLS and improve the efficacy of cancer immunotherapies. Clearly, further research is essential to better understand these complex mechanisms and to translate them into innovative, TLS‐targeted therapeutic approaches. Achieving this goal also requires better understanding of how TLS neogenesis and function are regulated within the tumor microenvironment by immune and nonimmune mechanisms. As already described for LNs, recent data have indicated that sensory and sympathetic nerves influence TLS formation and potentially affect the effector functions of immune cells expressing receptors for various neurotransmitters, such as dopamine and/or β2‐adrenergic receptors. The study of TLS interactions with the peripheral nervous system in cancer is still in its early stages, and further investigations are needed to understand the relationship between TLS and various types of nerve fibers (e.g., sympathetic, parasympathetic, and sensory) present in different tumor contexts. This endeavor first requires the use of preclinical animal models in which nerve fibers can be manipulated, and second, the investigation of human cancer biopsies and surgical pieces using technologies such as immunolabeling‐enabled imaging of solvent‐cleared organs (iDISCO) [155] and/or spatial biology. An attractive approach to decipher the function and regulation of human TLS may be the use of humanized mice, as discussed above. However, significant challenges remain before we can fully master the generation of TLS with antitumor activity in these models. Finally, the detection of TLS currently relies primarily on histological analyses performed directly on tumor tissue. However, the analysis of soluble biomarkers locally associated with TLS presence is an emerging and promising field. The coming years will reveal whether TLS will have a bright future as next‐generation immunotherapeutic drugs for cancer patients.

## Conflicts of Interest

M.‐C.D.‐N. and J.‐L.T. are named as inventors on several patents related to TLS (EP2013/051047, EU 3341732, WO PCT/EP2022/052984) and antibody engineering (EP 3187505 B1; EP1824887 B1). J.R. received personal funds from Union Française pour la Santé Bucco‐Dentaire (UFSBD). The other authors declare no conflicts of interest.

## Data Availability

No new data were generated for this review article.
